# The Needle of the Compass

**Published:** 1882-02

**Authors:** 


### THE NEEDLE OF THE COMPASS.

" I beg to state," said Prof. Patterson, of the coast survey, " that the reason why the needle points in the northerly direction, is that the earth in itself is a magnet, attracting the magnetic needle as the ordinary magnets do, and the earth is a magnet as the result of certain cosmical facts, much affected by the action of the sun. These laws have periodicities, all of which have not as yet been determined. The inherent and ultimate reason of any fact in nature, as gravity, light, heat, etc., is not known further than that it is in harmony with all facts in nature. Even an earthquake is in perfect harmony with, and the direct resultant of, the action of forces acting under general laws. A condensed explanation in regard to the needle pointing to the northward and southward, is as follows: The magnetic poles of the earth do not coincide with the geographical poles. The axis of rotation makes an angle of about 23 deg., with a line joining the former. The northern magnetic pole is at present near the Arctic Circle on the meridian of Omaha. Hence the needle does not everywhere point to the astronomical north, and is constantly variable within certain limits. At San Francisco it points about 17 degrees to the east of north, and at Calais, Me., as much to the west. At the northern magnetic pole, a balanced needle points with its north end downward in a plumb line. At San Francisco it dips about 63 degrees, and at the southern magnetic pole the south ends points directly down. The action of the earth upon a magnetic needle at its surface is of about the same force as that of a hard steel magnet, forty inches long, strongly magnetized, at a distance of one foot. The foregoing is the accepted explanation of the fact that the needle points to the northward and southward. Of course no ultimate reason can be given for this natural fact in nature."

### FARMERS' HOMES.

Mr. B. G. Northrup, in a useful lecture on " Farmers' Homes," called attention to suggestive statistics collected in Massachusetts, showing that the average age of 28,000 farmers at death was sixty-five years, and of 128,000 persons of all classes barely fifty-one years. It was found, however, that the wives and children of farmers are shorter-lived than the farmers themselves. Sanitary defects are among the chief causes of ill-health. Barnyards, hogpens, privies, cesspools, filters and cellars are far too prominent. Among women and children dyspepsia is a most frequent disease, and is induced by improper diet. Pies and cakes are too common on American tables. There should be more broiling and boiling, and less frying; more fresh and less salt meat. A high, dry location should be chosen for the dwelling, and even if it does cost more to dig the well, the water will be better than in the hard pan in the valley below. Wells should never be near to privies or cesspools, and the older the wells the greater the danger of impure water. The health of our grandparents is no guarantee that the wells they drank from still contain only pure water. Farmers stoop too much. Yankees are a race of stoopers, say some of our German and French cousins. Farming need not be a plodding, routine life; more pains should be taken with children at home; more books and periodicals should be provided, and they should be owned, not borrowed; reading aloud should be encouraged, and the art of conversation cultivated. Our educational system trains the intellect to the neglect of the sensibilities. The disparagement of country life has been one of the worst tendencies of the age. The love of home brings with it a love of country. Ownership in land is essential to thrift, and it gives bonds to society for maintaining a good character.

---

French authorities are investigating the subject of the influence of school-room arrangements upon the eyes of the pupils.

The preservation of articles of diet with salicylic acid has been prohibited by the French government, it being considered that this well-known preservative agent is dangerous to health.

The following is given as a specimen bill of fare at the dinner table of a Tennessee planter: Corn bread with only salt to season it, fat bacon and a dish of boiled turnip tops; for drink, strong black coffee made of the berries imported from Rio. This was all; no fruits, no preserves, no pastries of any kind. The trouble is that the planters give all their time to raising cotton.

Scene in a recitation room; arsenic under discussion. Pupil—"Arsenic possesses great beautifying properties, and many people take it to make themselves beautiful." Teacher—"I have taken arsenic, and I never observed that I grew beautiful under its effects." Pupil—"Well, of course, you have to have something to begin on."

